# Selection principles of polymeric frameworks for solid-state electrolytes of non-aqueous aluminum-ion batteries

**DOI:** 10.3389/fchem.2023.1190102

**Published:** 2023-04-11

**Authors:** Zhijing Yu, Yafang Xie, Wei Wang, Jichao Hong, Jianbang Ge

**Affiliations:** ^1^ State Key Laboratory of Advanced Metallurgy, University of Science and Technology Beijing, Beijing, China; ^2^ School of Metallurgical and Ecological Engineering, University of Science and Technology Beijing, Beijing, China; ^3^ School of Mechanical Engineering, University of Science and Technology Beijing, Beijing, China

**Keywords:** aluminum-ion batteries, solid-state polymer electrolytes, polymeric frameworks, chloroaluminate salts, ionic liquids

## Abstract

Liquid electrolyte systems of aluminum-ion batteries (AIBs) have restrictive issues, such as high moisture sensitivity, strong corrosiveness, and battery leakage, so researchers have turned their attention to developing high safety, leak-free polymer electrolytes. However, the stability of the active factor of AIB systems is difficult to maintain with most of polymeric frameworks due to the special Al complex ion balance in chloroaluminate salts. Based on this, this work clarified the feasibility and specific mechanism of using polymers containing functional groups with lone pair electrons as frameworks of solid-state electrolytes for AIBs. As for the polymers reacting unfavorably with AlCl_3_, they cannot be used as the frameworks directly due to the decrease or even disappearance of chloroaluminate complex ions. In contrast, a class of polymers represented by polyacrylamide (PAM) can interact with AlCl_3_ and provide ligands, which not only have no effect on the activity of Al species but also provide chloroaluminate complex ions through complexation reactions. According to DFT calculations, amide groups tend to coordinates with AlCl_2_^+^ *via* O atoms to form [AlCl_2_(AM)_2_]^+^ cations, while disassociating chloroaluminate anions. Furthermore, the PAM-based solid-state and quasi-solid-state gel polymer electrolytes were also prepared to investigate their electrochemical properties. This work is expected to provide new theoretical and practical directions for the further development of polymer electrolytes for AIBs.

## 1 Introduction

As global warming and environmental degradation caused by fossil energy become more and more serious, clean and green energy has attracted widespread attention (Liu et al., 2010; Chu and Majumdar, 2012; Gao et al., 2021). For the stable and flexible utilization of renewable green energy (such as wind, solar and tidal power), the development of sustainable electrochemical energy storage system is the most important step (Chu et al., 2017). The rechargeable aluminum-ion batteries (AIBs) have become one of the research hotspots of energy storage of energy storage due to the abundant resources and security feature of aluminum (Al) (Elia et al., 2016; Yang et al., 2019). In recent years, the research on AIBs mainly focuses on positive electrode materials, such as carbonaceous materials, oxides, elemental S/Se/Te and chalcogenides, as well as organic materials (Chen et al., 2017; Yang et al., 2018; Guo et al., 2020; Yu et al., 2020; Pan et al., 2022; Tu et al., 2022). And non-aqueous chloraluminate electrolyte systems are mostly employed, such as high-temperature molten salts, room temperature ionic liquids (ILs), etc (Song et al., 2017; Zhang et al., 2018). In 2015, the Al-C batteries with AlCl_3_/1-ethyl-3-methylimidazolium chloride ([EMIm]Cl) ILs as electrolyte were reported, marking a breakthrough in the research of AIBs (Lin et al., 2015; Sun et al., 2015). Since then, the room temperature chloroaluminate ILs have been basically applied as electrolyte systems of AIBs.

Room temperature chloroaluminate ILs (including ILs, IL analogues, etc.) have a series of advantages such as high ionic conductivity, strong electrochemical activity and good thermal stability (Armand et al., 2009). They provide chloroaluminate anions for the redox reaction of AIBs, with considerable electrochemical performance. However, their high moisture sensitivity would greatly limit the further practical application of AIBs (Huang et al., 2022). Even in the ambient environment, the chloroaluminate IL electrolytes are prone to absorb H_2_O, resulting in exothermic reaction to produce unfavorable gas, which will lead to battery leakage and distortion. These not only cause several critical problems for battery performance, including decreased Coulombic efficiency and irreversible capacity decay, but also may lead to corrosion of the parts of the equipment in practical applications, forming a certain potential safety hazard (Yu et al., 2019b).

Polymer electrolytes are leak proof and easily fabricated into the desired shapes and sizes, which makes the batteries are suitable for various geometries and pressure (Wang et al., 2013). More importantly, the polymer chains in the polymer electrolytes will encapsulate the active components as a protective framework from moisture (Liu et al., 2021a; 2021b). Meanwhile, polymer electrolyte is not only an ionic conductor but also an electronic insulator with certain mechanical strength, which could also serve as a separator. Thus, researchers have tried to develop the polymer electrolytes suitable for AIB systems and some progresses have been made (Yu et al., 2019b; Yu et al., 2019a; Liu et al., 2021a; Liu et al., 2021b). In these attempts, some uncommon polymers were employed as the polymeric frameworks, such as polyacrylamide (PAM) and polyamide (PA), for achieving gel electrolytes with chloroaluminate ILs as plasticizers. Nevertheless, the preparation principles of polymer electrolytes for AIBs, especially the selection of polymeric frameworks, has not been clearly studied and summarized.

Noticeably, for the polymer electrolytes of lithium-ion batteries (LIBs), the lithium salts provide the active factor Li^+^ to participate in the redox reaction during the charge and discharge process. But for non-aqueous AIBs, the active factors mainly exist in the form of chloroaluminate complex ions in the electrolyte. And the precondition for the reversible charge-discharge process of AIB is that the reversible deposition/dissolution reaction of Al can take place at the negative electrode, which further indicates the necessity of chloroaluminate complex ions for an AIB system (Tu et al., 2021). Unlike simple Li^+^, there is always an equilibrium between ion formation and decomposition for the chloroaluminate complex ions. Therefore, the active factor (chloroaluminate complex ions) in polymer electrolytes of AIBs could come from two ways: 1) Addition of liquid-state chloroaluminate electrolytes; 2) Generation by the coordination reactions between AlCl_3_ and the polymer matrix with active functional groups. That is, the polymers coexisting with liquid-state chloroaluminate electrolytes can be used to synthesize quasi-solid-state gel electrolytes, and the polymers meeting the second way can also be used to try all-solid-state electrolytes.

In this work, the selection principles of polymeric frameworks of solid-state electrolytes are developed for non-aqueous AIBs. The feasibility of several common polymers, including polyethylene oxide (PEO), polyacrylonitrile (PAN), polymethylmethacrylate (PMMA) and polyvinylidene fluoride (PVDF) are investigated. And they all have a repeating characteristic group with lone pair electrons that can be attached by Al atom in AlCl_3_, which has a certain impact on the activity of the chloroaluminate electrolyte systems. Why the polymers with amide group represented by PAM were selected as the polymeric frameworks of solid-state electrolytes of AIBs is also demonstrated in detail and density functional theory (DFT) calculations on the energy barriers provide insights into the complexation and dissociation process of chloroaluminate complex ions on the amide groups. This work will provide inspiration for further exploring advanced solid-state electrolytes of AIBs and promoting the practical device applications for energy storage.

## 2 Materials and methods

### 2.1 Materials

The polymers were purchased from Meryer (Shanghai) Biochemical Technology Co., Ltd. Dichloromethane (DCM, 99+%, Alfa Aesar), acrylamide (AM, 98.5%, Alfa Aesar), 1-ethyl-3-methylimidazolium chloride ([EMIm]Cl, 97%, Acros Chemicals), aluminum chloride (AlCl_3_, anhydrous, 99.999%, Sigma Aldrich), 2, 2′-azodiisobutyronitrile (initiator AIBN, >98%,TCI), were obtained from commercial sources. Platinum (Pt) wire and aluminum (Al) wire were received from General Research Institute for Non-ferrous Metals (Beijing, China).

### 2.2 Electrochemical and physical measurements

Al plating/stripping experiments: In the Al plating/stripping experiments, Pt and Al wires are used as working electrode and reference electrode, respectively, and the counter electrode is an aluminum coil. The cyclic voltammetry method is applied by CHI 1140C electrochemical workstation (Shanghai, China) with a scanning range of −0.6 to 1.0 V and a rate of 100 mV s^−1^.

Measurement of ionic conductivities: The ionic conductivities were analyzed by Electrochemical impedance spectroscopy (EIS) method in a Swagelok battery with two blocking Mo electrodes under the working temperatures from 20 to 100°C. EIS was carried out with a CHI 660E electrochemical workstation (Shanghai, China) at the frequency range of 100 kHz to 0.1 Hz.

The galvanostatic discharge and charge tests were performed by a multichannel battery testing system (Neware BTS-53) with different positive electrodes.

Raman spectroscopy (HORIBA, LabRAM HR Evolution) was performed to analyze ions information of electrolyte samples. The morphologies of the SPE samples and GPE were characterized by FE-SEM (JEOL, JSM-6701F) with an energy-dispersive X-ray spectrometer. FTIR spectra were applied by a NICOIET Fourier Transform Infrared Spectrometer.

### 2.3 Preparation of ILs and synthesis of PAM-based polymer electrolytes

Preparation of ILs: The room-temperature IL electrolyte was made by mixing [EMIm]Cl and anhydrous AlCl_3_ in an argon-atmosphere glovebox ([O_2_] <0.1 ppm, [H_2_O] <0.1 ppm). AlCl_3_ was slowly dissolved into [EMIm]Cl with constantly stirring, then the yellow transparent liquid could be obtained.

Synthesis of PAM-based polymer electrolytes: AM was slowly added to the dichloromethane suspension containing AlCl_3_, and a clear yellow liquid is obtained after two solid powders are completely dissolved. Then the initiator AIBN (1 *wt*% of AM) was added and the white solid-state polymerization product is obtained after solvent evaporation. The white solid sample obtained from polymerization reaction was ground into powder in an agate mortar. And an appropriate amount of the powder was taken and pressed into a diameter of 20 mm pellet under about 15 MPa force for 4 min, which was used for subsequent electrochemical testing. In addition, the GPEs were prepared by mixing 80 *wt*% ILs (based on total mass) before adding the initiator and the detailed preparation method has been thoroughly described in our previous work (Yu et al., 2019a).

### 2.4 Preparation of positive electrodes and assembly of quasi-solid-state Al batteries

Positive electrodes were prepared by fabricating the slurry of 70 *wt*% of active materials (graphite or Ni_3_S_2_, obtained from commercial sources), 20 *wt*% of acetylene black, and 10 *wt*% of binder in N-methyl-2-pyrrolidinone (NMP), followed by casting onto the Ta foil. The solid-state AIB was fabricated with an Al negative electrode, GPE and corresponding positive electrode into a pouch cell using Al laminate C8-480 and sealed with heat sealing machine (BLEUETS FR-300B) in the glove box.

### 2.5 First-principles calculations

All theoretical calculations were performed using density functional theory (DFT), as implemented in the DMol3 package (Delley, 1990; Perdew et al., 1996). The electron exchange and correlation energy were treated within the generalized gradient approximation in the Perdew-Burke-Ernzerhof functional (GGA-PBE). The global orbital cut-off energy for each element was set to 5.2 Å. The convergence criteria for the electronic self-consistent iteration and force were set to 10^−5^ Ha and 0.004 Ha Å^−1^, respectively. The choice of these computational parameters ensured good convergence in the present studies.

## 3 Results and discussion

**FIGURE 1:**
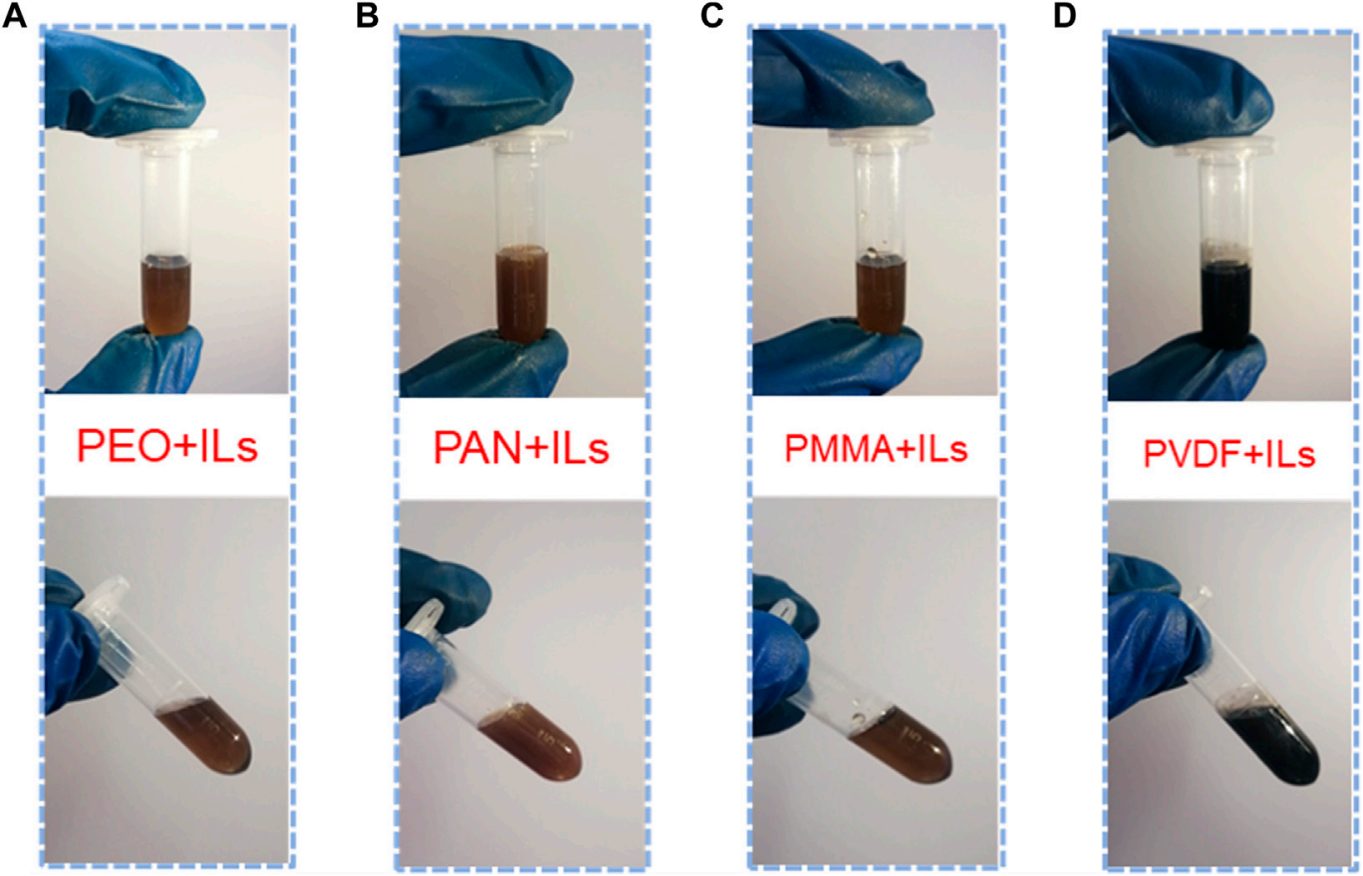
Photos of the polymer (5 *wt*%)-IL mixtures **(A)** PEO. **(B)** PAN. **(C)** PMMA. **(D)** PVDF.

The chemical and electrochemical stability of PEO, PAN, PMMA, and PVDF were first studied when the chloroaluminate complex ions were directly supplied by adding AlCl_3_/[EMIm]Cl IL electrolytes. As shown in Figure 1, different polymers with 5% mass fraction (based on the mass of ILs) were added to the ILs, respectively. It can be observed that the mixtures of different polymers and ILs still present a flowing liquid state at room temperature, indicating that the adding amount of polymer matrix is far from enough to prepare a self-supporting electrolyte membrane (Supplementary Figure S1). In particular, when PVDF was added, the ILs change from transparent yellow to black, and release a lot of heat during the mixing process.

**FIGURE 2:**
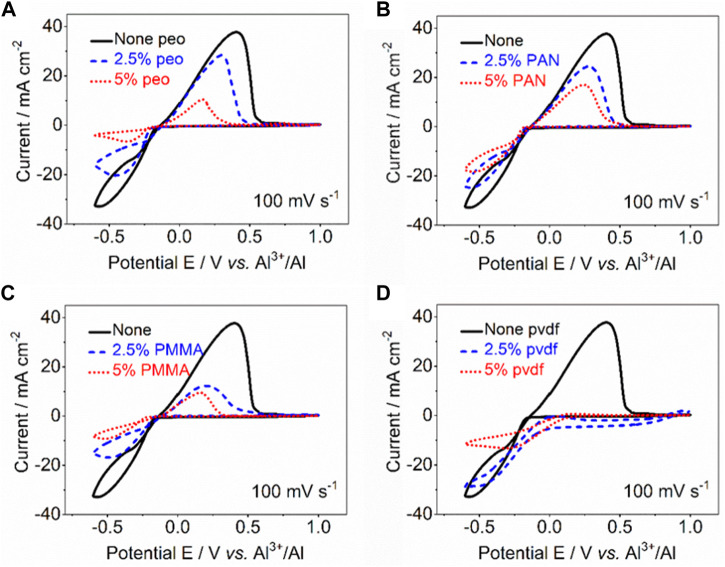
CV curves for determining the Al plating/stripping in IL mixtures with different ratio of polymers **(A)** PEO. **(B)** PAN. **(C)** PMMA. **(D)** PVDF.

The effect of different adding amounts of polymers on the electrochemical deposition behavior of Al was investigated to determine whether the reversible reaction on the negative electrode of AIBs could occur in the mixtures. Figure 2 shows the typical cyclic voltammetry (CV) curves within the voltage range of −0.6∼1.0 V at a rate of 100 mV s^−1^. The black solid line represents the CV curve without adding polymer, and there is a pair of obvious redox peaks at −0.6 and 0.4 V, corresponding to the plating and stripping of Al. However, the redox peak currents decrease with the increase of polymer content, suggesting that addition of the polymers will have a negative impact on the reversible reaction. To make matters worse, the oxidation peaks almost disappear after PVDF added, indicating that this system is most affected. Noticeably, the reversible Al plating/stripping reactions occur mainly due to the formation of electrochemical active substance Al_2_Cl_7_^−^ in the presence of excessive AlCl_3_ in acidic AlCl_3_/[EMIm]Cl IL electrolytes. Based on this, the acidic AlCl_3_/[EMIm]Cl ILs (at a mole ratio of 1.3) can be used as electrolyte for AIBs. However, the activity of the electrolytes decreases after adding the four kinds of polymers of PEO, PMMA, PAN and PVDF, which can be preliminarily inferred that the amount of Al_2_Cl_7_^−^ is also greatly reduced.

**FIGURE 3:**
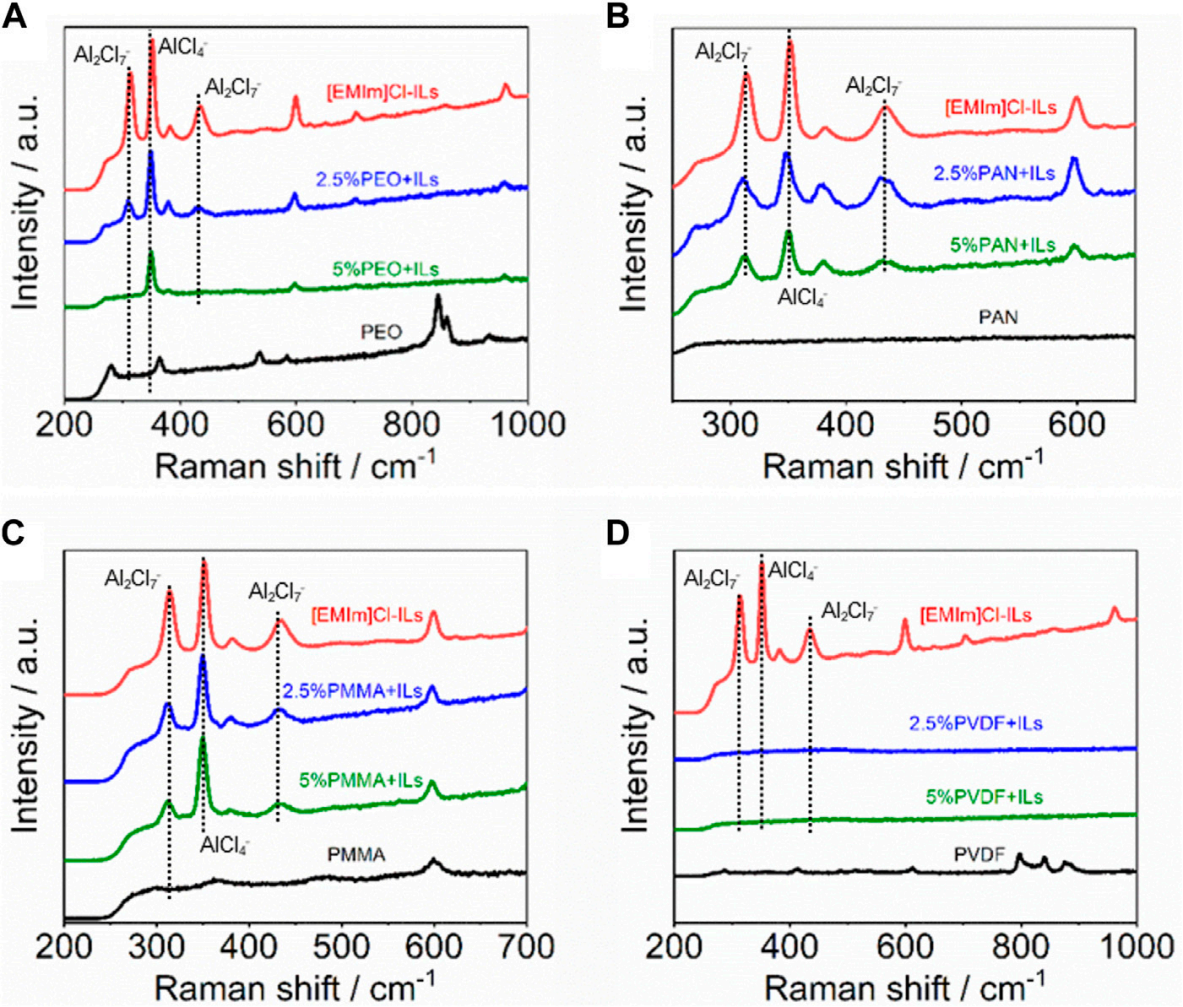
Raman spectra of the mixtures with different ratio of polymers and ILs **(A)** PEO. **(B)** PAN. **(C)** PMMA. **(D)** PVDF.

In order to study the change of the occurrence form of chloroaluminate complex ions, Raman spectra of polymer/IL mixtures with different proportions were tested respectively, as shown in Figure 3. The signals of AlCl_4_^−^ (350 cm^−1^) and Al_2_Cl_7_^−^ (311 and 432 cm^−1^) were detected in the original ILs without polymers (Xu et al., 2019). When 2.5% of PEO is added, the characteristic peak intensity of AlCl_4_^−^ and Al_2_Cl_7_^−^ are significantly weakened, and when the amount is increased to 5%, the signal of Al_2_Cl_7_^−^ almost disappears (Figure 3A). It shows that the chloroaluminate complex ions are consumed with the addition of PEO and the electrochemical activity of the IL electrolytes is greatly reduced. From the comparison in Figures 3B, C, the similar decreasing trend of chloroaluminate complex ions, especially Al_2_Cl_7_^−^, also can be seen in the mixtures with increasing addition of PAN and PMMA. After adding PVDF, almost no signal peaks are observed, indicating that the reaction between PVDF and ILs is more intense and the system is completely deactivated. Fourier transform infrared (FT-IR) spectra were used to analyze the functional groups in polymer/IL mixtures with different proportions (Supplementary Figure S2). The absorption peak of polymers is difficult to distinguish in the mixtures, which may be due to insufficient polymer addition or structural change. However, the asymmetric vibration of Al-Cl bonds located at 492 cm^−1^ tends to disappear with the increase of polymer addition, which further proves the decrease of electrochemical activity.

In the chloroaluminate electrolytes, the proportion of AlCl_3_ determines that the system is basic, neutral, or acidic. And both AlCl_4_^−^ and Al_2_Cl_7_^−^ co-exist in the acidic electrolyte system. For the formation of AlCl_4_^−^ and Al_2_Cl_7_^−^ anions, there is the following reactions (Melton et al., 1990; Tu et al., 2021):

$${AlCl}_{3}{+Cl}^{-}⇌{AlCl}_{4}^{-}$$ (1)

$${AlCl}_{4}^{-}{+AlCl}_{3}⇌{Al}_{2}{Cl}_{7}^{-}$$ (2)

$${2AlCl}_{4}^{-}⇌{Al}_{2}{Cl}_{7}^{-}{+Cl}^{-}$$ (3)

Among them, the outermost layer of the central atom Al of AlCl_3_ has an empty orbit. To achieve the 8-electron stable structure, Al atom in AlCl_3_ is easy to accept the lone pair electrons to form a special covalent bond, that is, coordination bond. The structural unit of PEO has a characteristic ether oxygen bond, and the oxygen atom in it has the ability to provide lone pair electrons (Supplementary Figure S3A). It combines with the AlCl_3_ in chloroaluminate electrolytes, which causes the ion balance of Eqs 1–3 move to the left. Similarly, the nitrogen atom in PAN structure, the oxygen atom in PMMA structure and the fluorine atom in PVDF structure can provide the lone pair electrons to react with AlCl_3_, resulting in the decrease or even disappearance of chloroaluminate complex ions (Supplementary Figures S3B–D). The structural unit of PVDF has a strong polar group of CF_2_, which is easier to provide lone pair electrons, so its reaction with AlCl_3_ is more intense, even leading to complete deactivation of the electrolyte system. Thus, it can be concluded that this type of polymers is not a suitable framework for solid-state electrolytes of AIBs. To further study the feasibility of PEO, PAN and PMMA, they were mixed into dichloromethane suspension containing AlCl_3_, respectively. After mixing, the color of the mixing samples changed from transparent and colorless to different shades of yellow, indicating that they already contain the reaction products of polymer and AlCl_3_ (Supplementary Figure S4A). Supplementary Figure S4B shows the Raman spectra of the samples and AlCl_3_/[EMIm]Cl ILs. Obviously, the reaction of PEO, PAN and PMMA with AlCl_3_ did not produce chloroaluminate complex ions. Therefore, PEO, PAN, PMMA and PVDF are not the best choice for directly using as polymeric frameworks of solid-state electrolytes for AIBs. So, why can polymers such as PAM be used?

**FIGURE 4:**
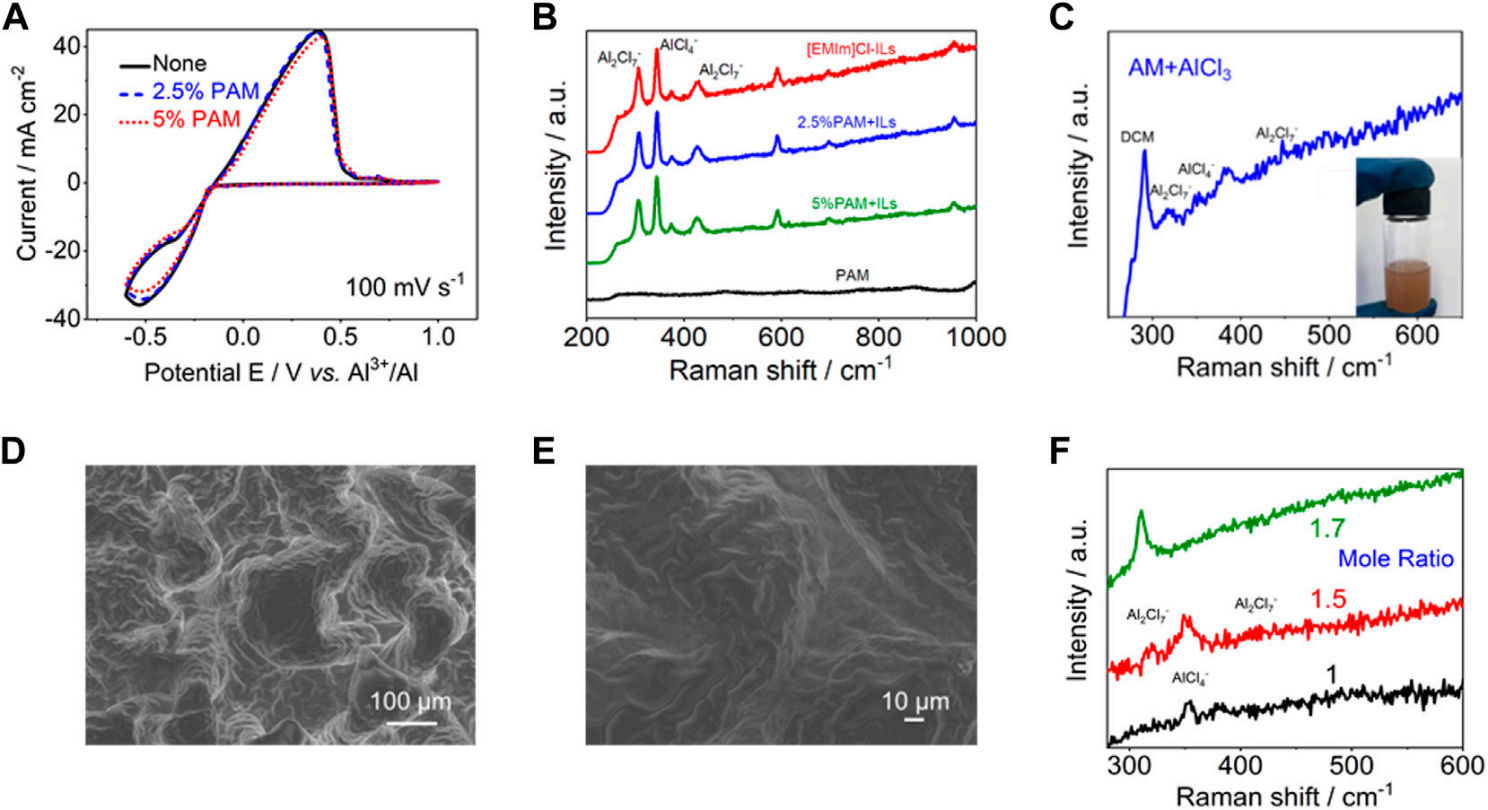
**(A)** CV curves for determining the aluminum plating/stripping in IL mixtures with different ratio of PAM. **(B)** Raman spectra of the mixtures with different ratio of PAM and ILs. **(C)** Raman spectrum of AlCl_3_/AM mixture. **(D,E)** SEM images of the polymerized solid-state sample. **(F)** Raman spectra of polymerization products at different AlCl_3_:AM mole ratios.

In order to verify the feasibility of PAM as polymeric framework for Al-based electrolyte system, the deposition/dissolution behavior of Al in the mixtures of AlCl_3_/[EMIm]Cl ILs with different mass fractions of PAM was investigated by CV tests, as shown in Figure 4A. With the addition of PAM, the shape of CV curves almost coincides with that in pure ILs, indicating that PAM has no effect on the electrochemical activity of chloroaluminate. Meanwhile, there is no significant reduction of chloroaluminate complex ions in Raman spectra of the mixtures, showing chemical stability (Figure 4B). To further study the Lewis acid-base interaction between amide groups in PAM structure and AlCl_3_, acrylamide (AM) and AlCl_3_ were dissolved in dichloromethane solvent. Interestingly, the signals of AlCl_4_^−^ and Al_2_Cl_7_^−^ can be observed from the Raman spectrum of the mixed solution (Figure 4C). After polymerization with the initiator, it appears as a white solid without any flow sign (Supplementary Figure S5). And the scanning electron microscopy (SEM) images of the solid-state sample are shown at different magnifications in Figures 4D, E. When AlCl_3_ and amide with different molar ratios are applied to prepare the precursor solutions, the occurrence forms of chloroaluminate complex ions in the polymerization products will also have some differences. As shown in Figure 4F, the Raman spectra of the polymerization products obtained at AlCl_3_: AM molar ratio of 1, 1.5 and 1.7 respectively. The results show that AlCl_4_^−^ and Al_2_Cl_7_^−^ anions coexist at a molar ratio of 1.5, which basically meets the requirement for Al-based energy storage electrolytes in terms of ionic structures.

**FIGURE 5:**
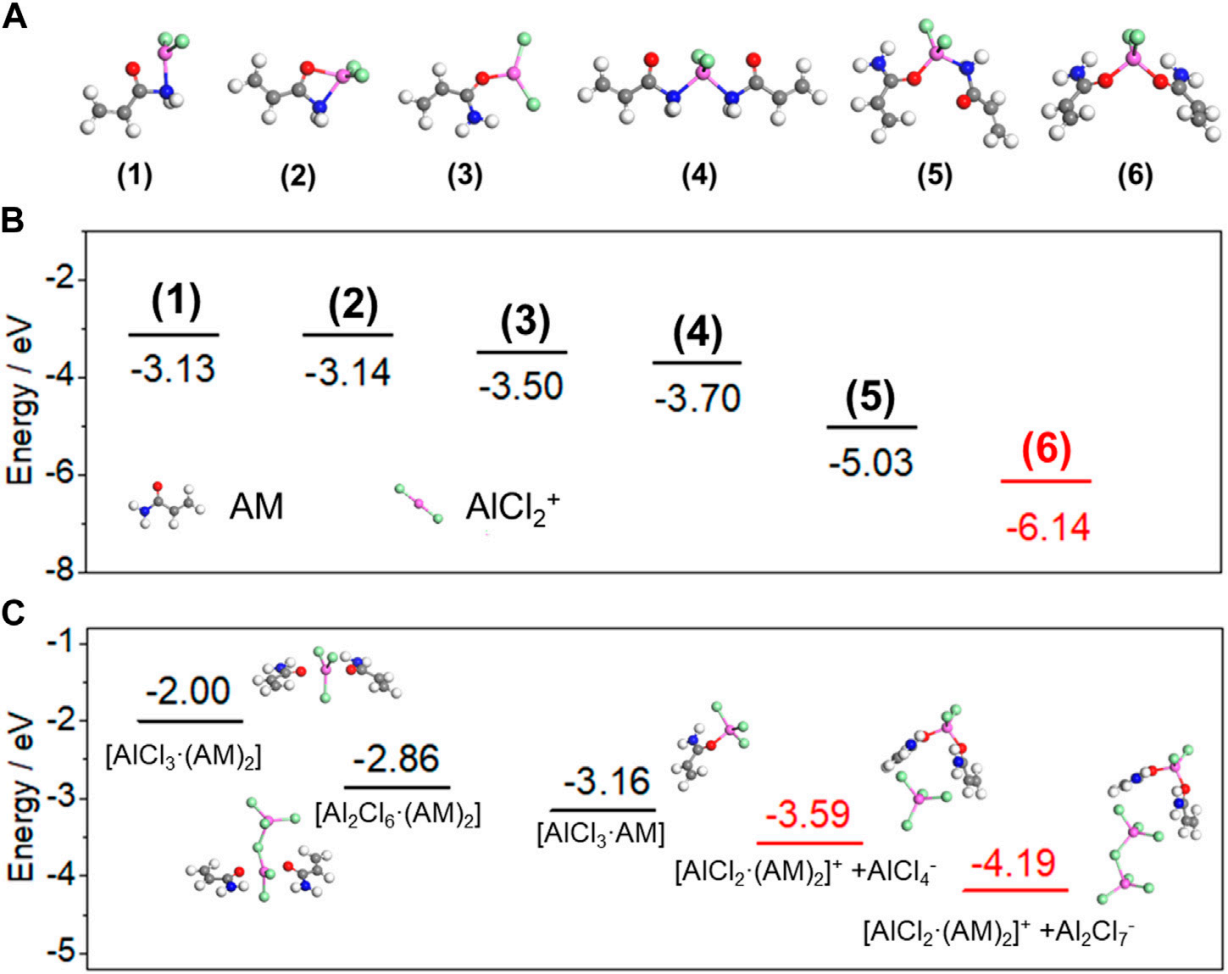
**(A)** Relaxed structures of the complex models based on different coordination sites. **(B)** Energy profiles of AM coordinating with AlCl_2_^+^ through different sites. **(C)** Energy profiles of five constructed models for coordination products. (Al: pink; Cl: green; O: red; N: blue; C: gray; H: white).

Besides the experimental methods, DFT calculations were also adopted to analyze the coordination structures and ion dissociation processes between polymer monomer AM and AlCl_3_. From Raman results, chloroaluminate anions will be generated when AM coordinates with AlCl_3_. To balance the electrical neutrality, Al-containing cations in the form of [AlCl_2_(AM)_n_]^+^ (*n* = 1 and 2), must exist due to the molecule nature of AM (Abood et al., 2011; Paterno et al., 2020). Thus, six complex models were established based on different coordination sites between AM and AlCl_2_^+^ (Figure 5A; Supplementary Table S1). As shown in Figure 5B, the [AlCl_2_(AM)_2_]^+^ structure formed by coordination *via* Al atom and two of O atoms is exothermic with a binding energy of −6.14 eV. In contrast, the structures based on other coordination sites are less stable. Therefore, AM tends to coordinates with AlCl_2_^+^ *via* O atoms to form [AlCl_2_(AM)_2_]^+^ cations. Furthermore, the ion dissociation in the complex reaction is also confirmed and five constructed models of coordination products were taken into account. The relaxed structures of the above five models and their calculated total energies are presented in Supplementary Figure S6 and Supplementary Table S2. Based on the dissociation energy study, the processes for dissociating chloroaluminate complex ions is more kinetically preferred with the more negative binding energies (Figure 5C).

**FIGURE 6:**
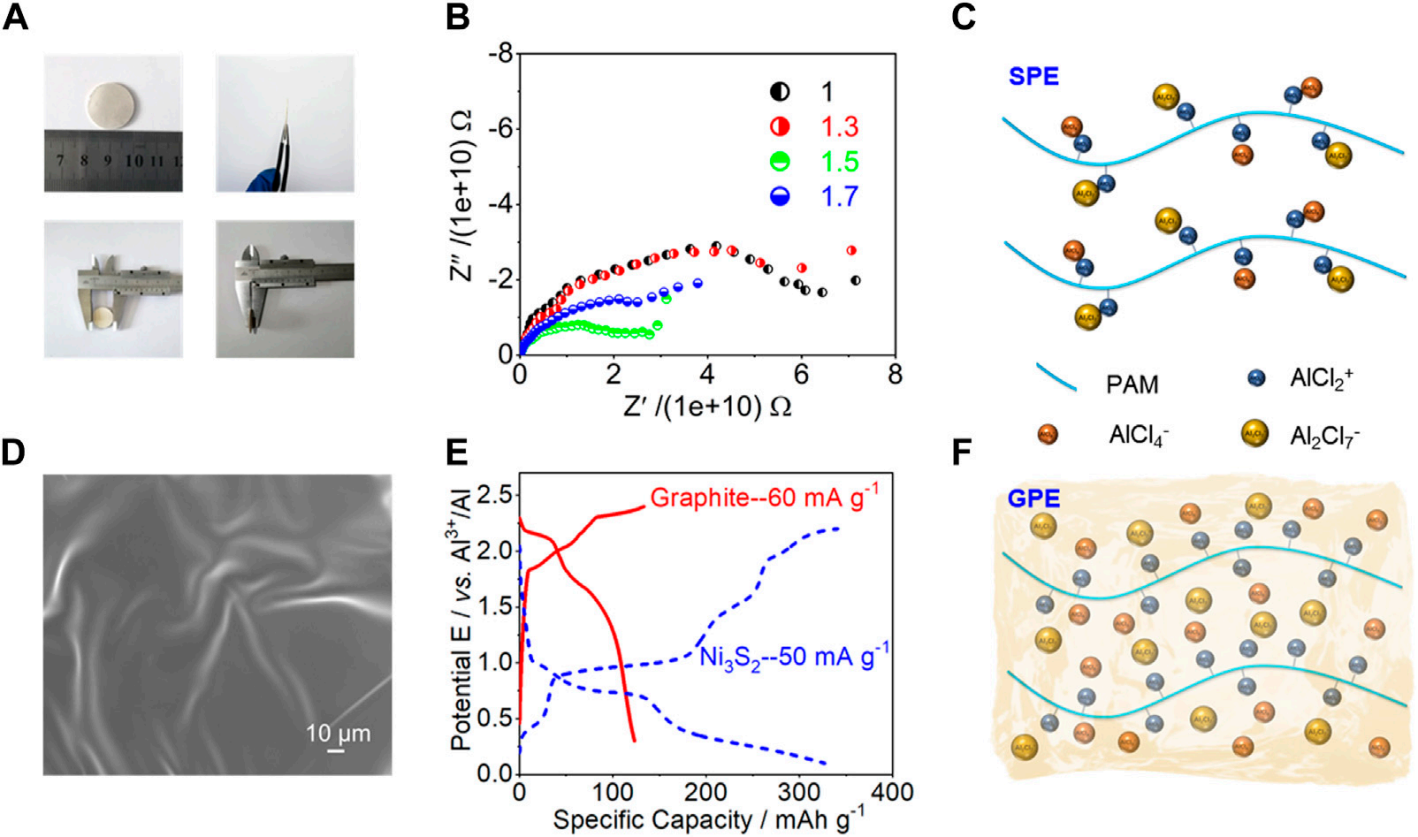
**(A)** Optical photos of the PAM-based SPEs obtained after pressing. **(B)** Nyquist impedance plots of Swagelok model cells based on the SPEs with different AlCl_3_:AM mole ratios. **(C)** Scheme for illustrating ion transport in PAM-based SPEs. **(D)** SEM image of GPE sample. **(E)** Charge and discharge curves of quasi-solid-state Al batteries based on different positive electrodes and GPE. **(F)** Scheme for illustrating ion transport in PAM-based GPEs.

Figure 6A shows the optical photos of the PAM-based solid-state polymer electrolytes (SPEs) obtained after pressing. The diameter, thickness and shape of the electrolyte can be controlled to optimize battery performance by changing the pressing conditions. The circular SPE shown in Figure 6A has a diameter of 20 mm and a thickness of 0.5 mm, which could be applied to many applications. In the Swagelok Mo/SPE/Pt cells (Supplementary Figure S7), AC impedance method was used to analyze the ionic conductivities of the SPEs at room temperature. Figure 6B exhibits the typical impedance spectra of the SPEs with different AlCl_3_: AM molar ratios (1, 1.3, 1.5 and 1.7), consisting of a semicircle in the mid to high frequency region and a straight line in the low frequency region. The corresponding ionic conductivities of the SPEs were determined by (Kuo et al., 2003):

$$\sigma =R_{b}^{-1}S^{-1}d$$ (4)

where *σ* is ionic conductivity, *R*_*b*_ the bulk impedance, *S* the geometric area of the electrolyte-electrode interface, and *d* the thickness. According to the Nyquist plots, the SPEs show very low conductivities, suggesting that the ion transport on the polymer chains is very difficult for the large radius chloroaluminate complex ions (Figure 6C). It may be due to factors such as the high impedance of the electrode-electrolyte interface, or the incomplete dissociation of ions in the solid state.

To achieve higher ionic conductivity, the ILs were added as plasticizers to form gel polymer electrolyte (GPE) membranes. Then the GPE sample surface tends to be uniform, smooth and flexible with addition of IL plasticizers (Figure 6D, Supplementary Figure S8). Obviously, the ionic conductivity will be raised with increasing the IL amount. When the content of ILs increases to 90%, it will lead to liquid behavior and cannot form a self-supporting electrolyte membrane (Supplementary Figure S9). Poor mechanical properties of the GPEs may cause a short circuit in the battery, thus affecting its use in energy applications. Therefore, the content of ILs was chose as 80% to prepare a self-supporting GPE membrane. As can be seen from Figure 6E and Supplementary Figure S10, excellent electrochemical performance can be obtained by applying the gel electrolyte, even if the batteries were assembled with positive electrode materials based on different energy storage mechanisms. Meanwhile, it can be observed in Supplementary Figure S11 that the fully charged quasi-solid-state Al batteries (one for graphite positive electrode and two for Ni_3_S_2_ positive electrode) based on different positive electrodes could power up a light-emitting-diode (LED, working voltage: 1.8 V) lamp. Therefore, ILs are introduced into solid-state electrolytes, turning polymer electrolytes from solid to gel-state, with the dual advantages of liquid and solid-state electrolytes. While the ionic liquid acts as a plasticizer, it also serves as an ion source to increase the concentration of charge carriers in the system, thereby improving the conductivity of the electrolyte (Figure 6F).

## 4 Conclusion

In conclusion, the preparation principles of polymer electrolytes for AIBs and the selection of polymer frameworks were proposed. Polymers that contain functional groups with lone pair electrons can be divided into two categories. One reacts unfavorably with AlCl_3_, resulting in a reduction or disappearance of chloroaluminate complex ions, such as PEO, PAN, PMMA and PVDF. They cannot directly serve as the framework of solid-state electrolytes for AIBs and special control measures need to be taken during the preparation process, such as conducting polymerization reactions based on AlCl_3_-free electrolyte or using blocking agents, to ensure that the activity of Al species is not affected. The other undergoes complexation reactions with AlCl_3_, which can provide active factor (chloroaluminate complex ions). An example of such a polymer is PAM, and it can be used to prepare an all-solid-state electrolyte. However, due to the difficulty in transferring large-radius chloroaluminate complex ions, it is necessary to add ionic liquids as plasticizers to improve ionic conductivity and form a gel electrolyte. Another feasible option is to select polymer frameworks that do not contain functional groups with lone pair electrons, which do not react with AlCl_3_, thus maintaining the stability of the active Al species. Therefore, this work can provide guidance for choosing appropriate polymer frameworks in preparing solid-state electrolytes of AIBs.

## Data Availability

The original contributions presented in the study are included in the article/Supplementary Material, further inquiries can be directed to the corresponding author.
