# Supplementary material for: Selection principles of polymeric frameworks for solid-state electrolytes of non-aqueous aluminum-ion batteries
Source: Front Chem. 2023 Apr 11;11:1190102. doi: 10.3389/fchem.2023.1190102 (PMC10126392; doi:10.3389/fchem.2023.1190102)
Supplement: Supplementary file 1 [file DataSheet1.docx]

Supplementary Material

Selection principles of polymeric frameworks for solid-state electrolytes of non-aqueous aluminum-ion batteries

Zhijing Yu^1,2^, Yafang Xie^2^, Wei wang^1,2^, Jichao Hong^3^, Jianbang Ge^1*^

^1^ State Key Laboratory of Advanced Metallurgy, University of Science and Technology Beijing, Beijing 100083, China.

^2^ School of Metallurgical and Ecological Engineering, University of Science and Technology Beijing, Beijing 100083, China.

^3^ School of Mechanical Engineering, University of Science and Technology Beijing, Beijing 100083, China.

*** Correspondence:** Jianbang Ge: jianbangge@ustb.edu.cn


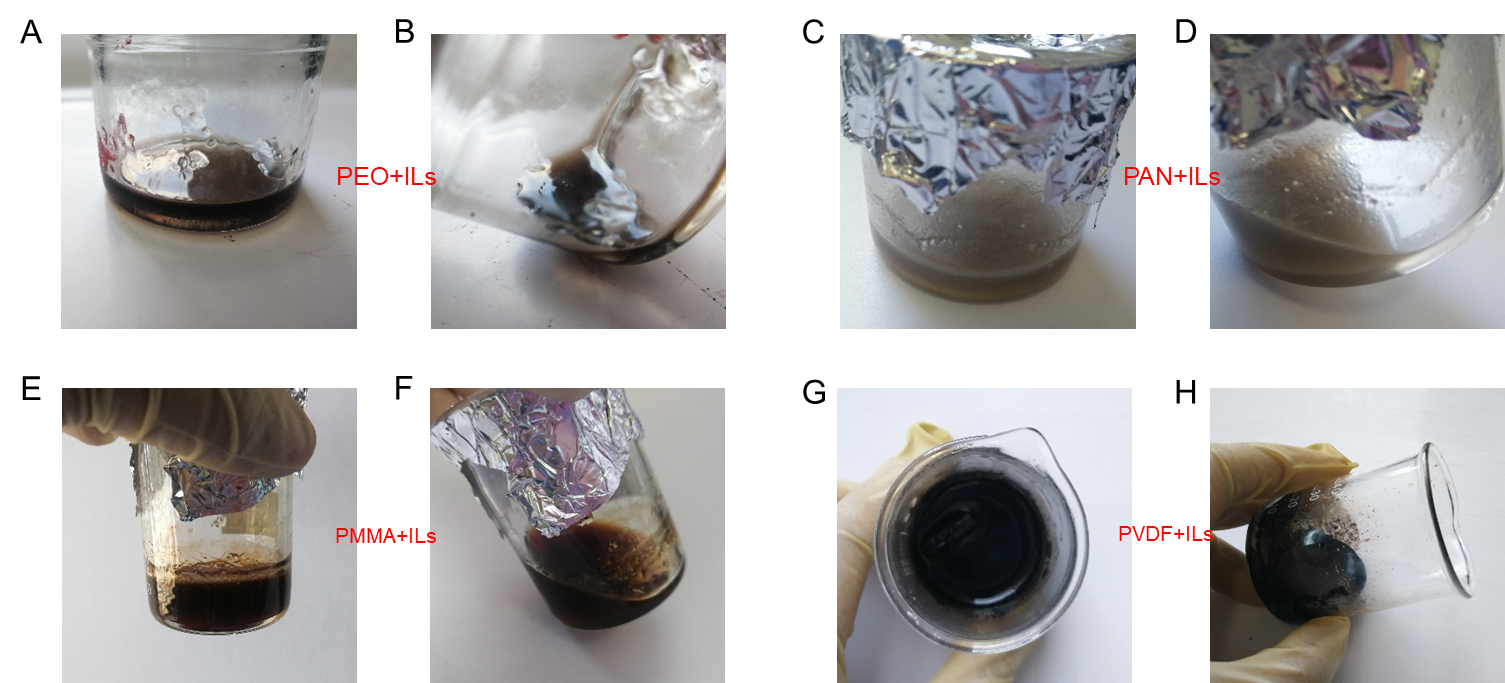


**Supplementary Figure 1.** Photos of the polymer/IL mixtures (A, B) PEO. (C, D) PAN. (E, F) PMMA. (G, H) PVDF

**
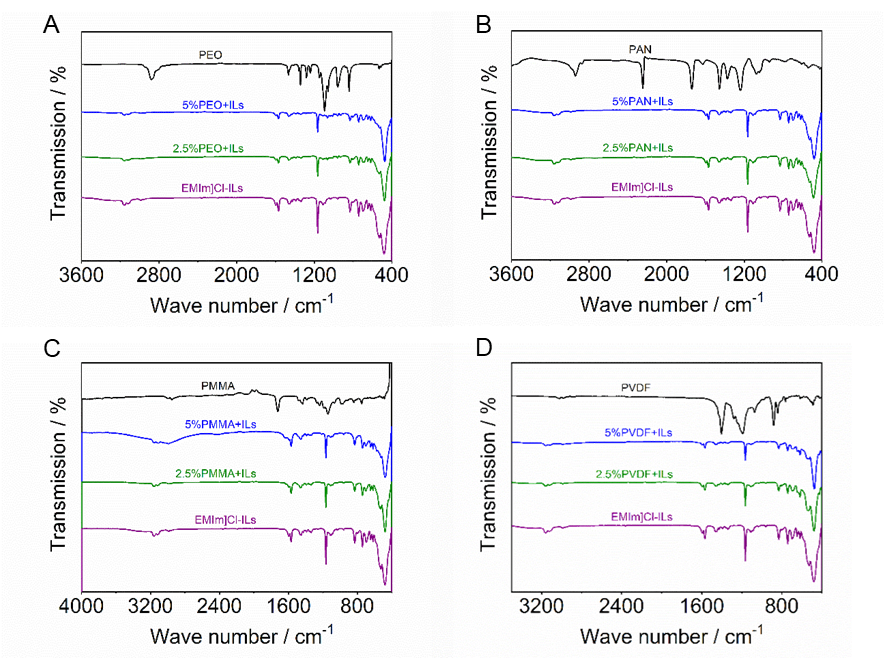
**

**Supplementary Figure 2.** FTIR spectra of the mixtures with different ratio of polymers and ILs (A) PEO. (B) PAN. (C) PMMA. (D) PVDF.

**
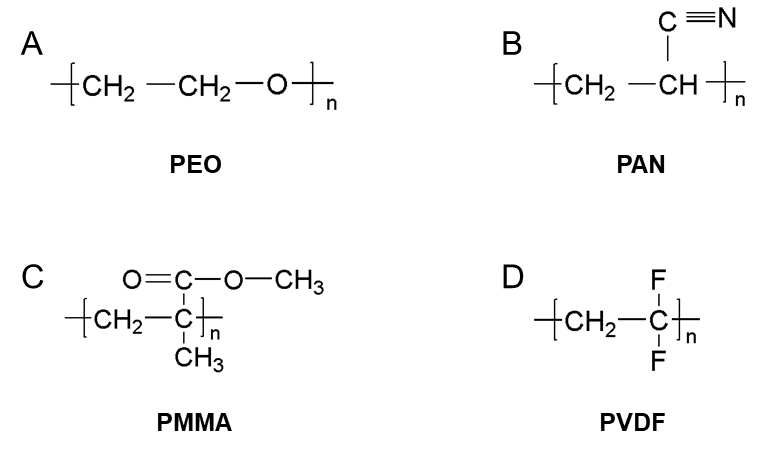
**

**Supplementary Figure 3.** Chemical structure of the polymers (A) PEO. (B) PAN. (C) PMMA. (D) PVDF.

**
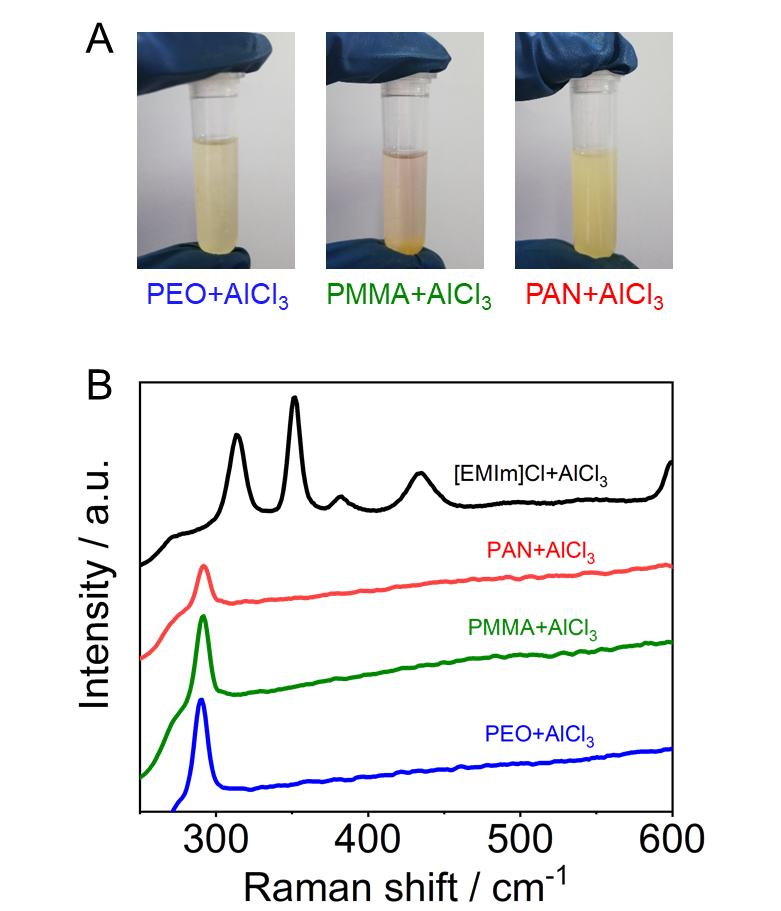
**

**Supplementary Figure 4.** Analysis of reaction products (a) Photos. (b) Raman spectra.

**
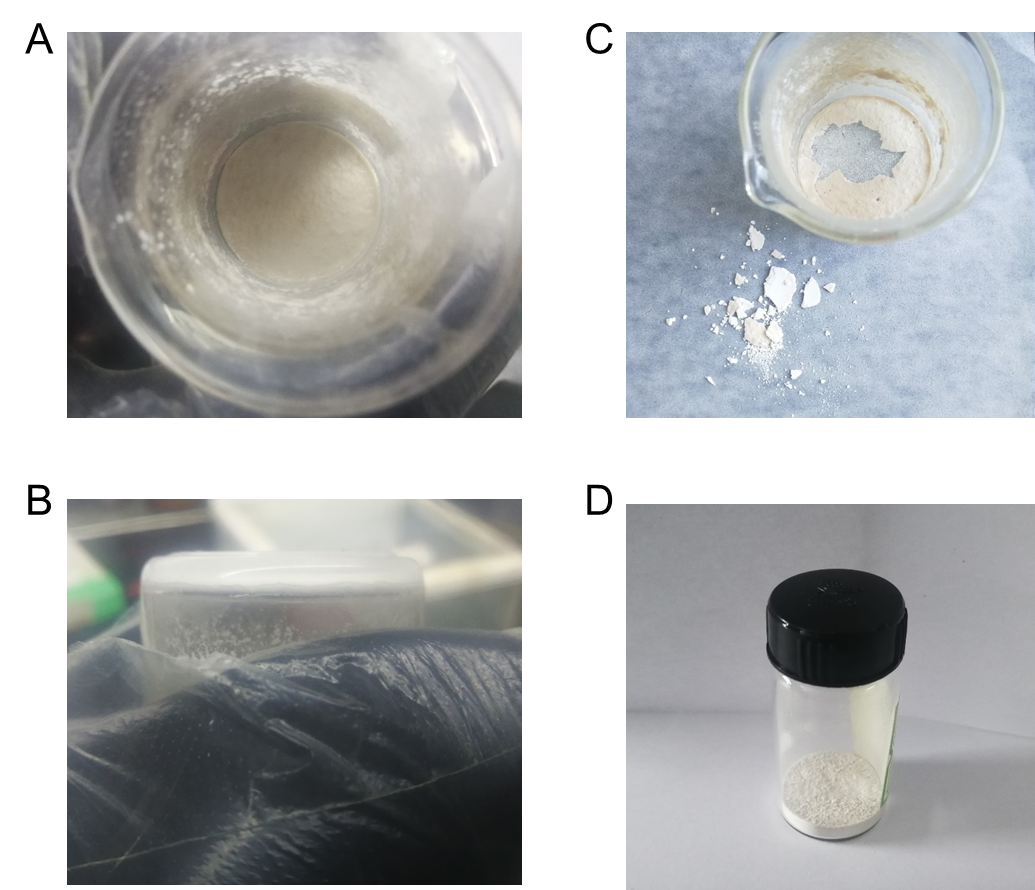
**

**Supplementary Figure 5.** Polymerization solid-state products.


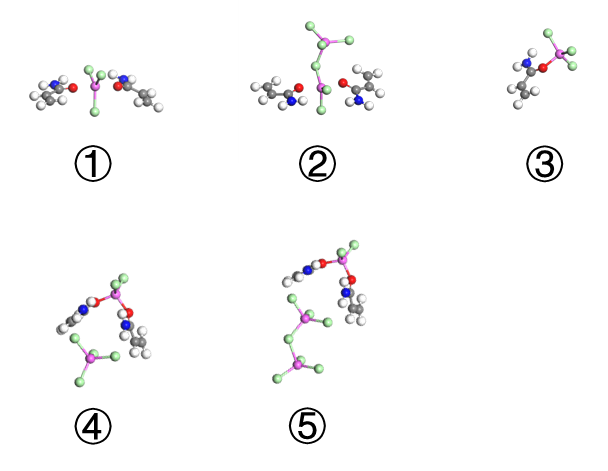


**Supplementary Figure 6.** Relaxed structures of five constructed models for coordination products (Al: pink; Cl: green; O: red; N: blue; C: gray; H: white).


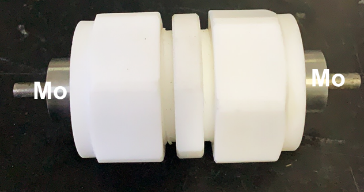


**Supplementary Figure 7.** Swagelok battery with Mo electrodes.


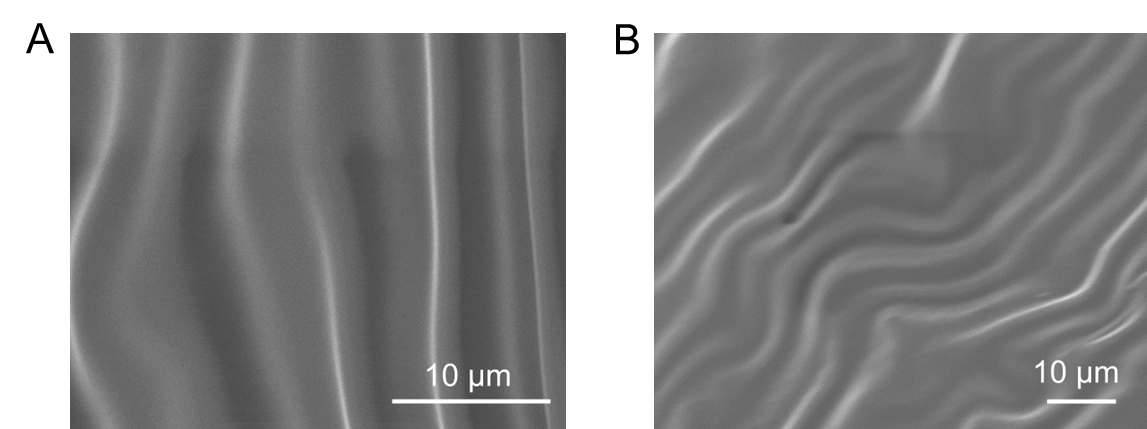


**Supplementary Figure 8.** SEM images of the GPEs with 80% ILs.


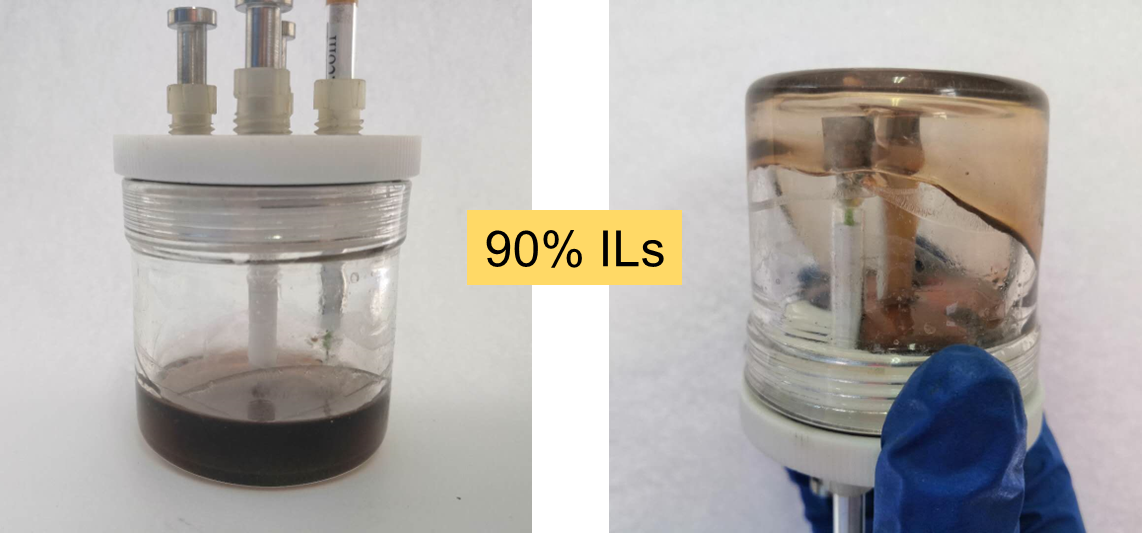


**Supplementary Figure 9.** Photos of the GPEs with 90% ILs.


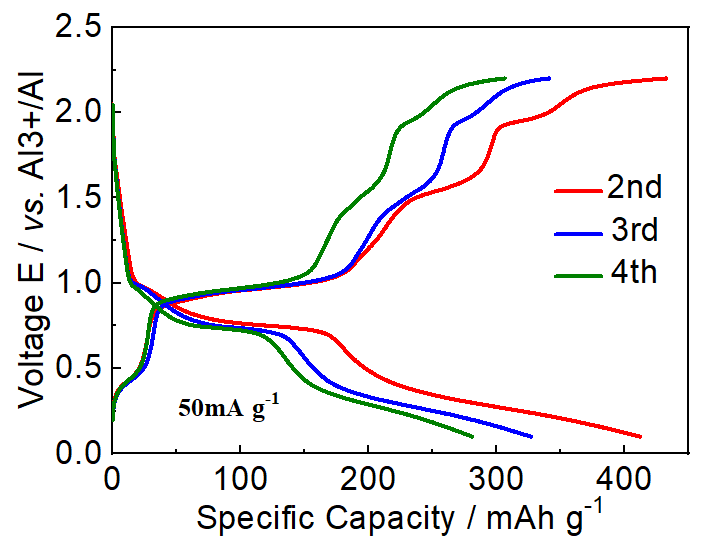


**Supplementary Figure 10.** Charge and discharge curves of Al-Ni_3_S_2_ battery with GPEs.

**
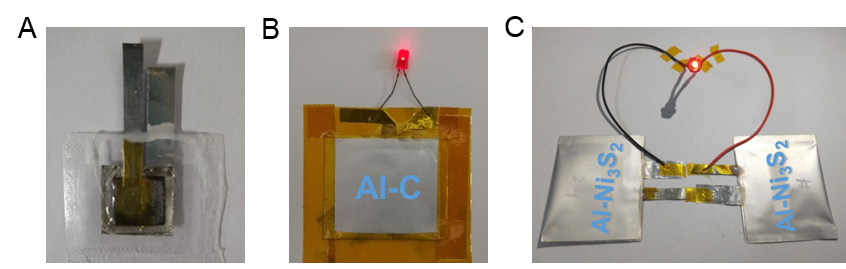
**

**Supplementary Figure 11.** (A) Photos of the quasi-state pouch cell. (B, C) Fully charged quasi-state Al battery powering up a LED lamp (working voltage: 1.8 V).

**Supplementary Table 1.** Calculated total energies of the corresponding complex models based on different coordination sites.

|  | Total energy / eV |  | Total energy / eV |
| --- | --- | --- | --- |
| (1) | -38353.09809 | (2) | -38353.09907 |
| (3) | -38353.46056 | (4) | -45077.96336 |
| (5) | -45079.29013 | (6) | -45080.39835 |

**Supplementary Table 2.** Calculated total energies of the corresponding complex models based on different coordination sites.

|  | Total energy / eV |  | Total energy / eV |
| --- | --- | --- | --- |
| ① | -57605.80932 | ② | -101761.8856 |
| ③ | -50881.09355 | ④ | -101762.6164 |
| ⑤ | -145918.4303 |  |  |
